# Prodromal behavioral markers and developmental trajectories of autism spectrum disorder in infancy: a narrative review

**DOI:** 10.3389/fped.2026.1788230

**Published:** 2026-03-06

**Authors:** Zifo Fang, Xiaoke Zhao

**Affiliations:** Department of Rehabilitation, Children’s Hospital of Nanjing Medical University, Nanjing, China

**Keywords:** autism spectrum disorder, developmental cascade, developmental trajectories, digital phenotyping, early screening, infancy, prodromal markers

## Abstract

Autism Spectrum Disorder (ASD) is a neurodevelopmental condition characterized by social communication deficits and restricted, repetitive behaviors. Despite the rising global prevalence, a significant gap remains between the biological onset of the disorder and the average age of clinical diagnosis (3–5 years). This “diagnostic lag” hinders access to early intervention during the critical window of neuroplasticity. Here, we synthesize recent evidence regarding behavioral markers in the prodromal phase (0–12 months) and the symptom consolidation phase (12–24 months), with a specific focus on the heterogeneity of developmental trajectories. Unlike static disease models, ASD manifests through dynamic “developmental cascades.” During the prodromal phase, non-specific signs—such as motor delays, attentional disengagement, and sensory regulatory issues—often precede overt social deficits. By the second year, these early vulnerabilities cascade into core symptoms, including the loss of joint attention, diminished response to name, and the emergence of restricted and repetitive behaviors. We further distinguish between “early-onset” and “regressive” patterns. Ultimately, by integrating empirical evidence with emerging digital phenotyping, we advocate shifting the clinical paradigm from “waiting for diagnosis” to “monitoring developmental trajectories,” thereby optimizing early identification strategies to improve long-term outcomes for affected children and their families.

## 1 Introduction

Autism Spectrum Disorder (ASD) is a complex neurodevelopmental condition characterized by persistent deficits in social communication and social interaction, alongside restricted, repetitive patterns of behavior, interests, or activities (1). The global prevalence of ASD has risen significantly in recent decades. According to the latest data from the U.S. Centers for Disease Control and Prevention (CDC), the prevalence among 8-year-old children has reached approximately 1 in 36 (2.8%) (2, 3). Despite increasing public awareness, the phenomenon of “diagnostic lag” remains severe: although parents often report concerns regarding developmental anomalies within the first year of life, the average age of clinical diagnosis globally persists between 3 and 5 years (4). This delay hinders access to early intervention during the critical window of peak neuroplasticity. However, delaying action until a definitive diagnosis is increasingly viewed as unnecessary. Current clinical evidence supports two key rationales for earlier engagement: First, intervention models are shifting toward “symptom-based” support, where therapeutic strategies address specific functional deficits, such as social communication delays, independent of a diagnostic label (5). Second, contrary to concerns about misdiagnosis, recent longitudinal studies demonstrate that ASD diagnoses made by experienced clinicians in the second year (12–24 months) are highly stable, exceeding 80%, with a low rate of false positives (6, 7).

Given the reliability of early detection and the benefits of early support, addressing this diagnostic lag requires a paradigm shift toward identifying the earliest precursors (5). Yet, a critical distinction must be drawn between the non-specific prodromal phase (0–12 months) and the symptom consolidation phase (12–24 months). Current evidence suggests that behavioral markers of ASD evolve dynamically rather than appearing statically. During the prodromal phase in the first year of life (0–12 months), infants typically do not exhibit overt social deficits. Instead, they may present with subtle, non-specific deviations in motor development, sensory processing, and attentional disengagement, which are often precursors to later social impairment (8). By the second year (12–24 months), as social demands increase, these subtle deviations gradually “cascade” into the distinct behavioral phenotype of ASD—such as the loss of joint attention and the emergence of restricted and repetitive behaviors (RRBs)—making reliable clinical diagnosis possible (9).

Understanding the developmental trajectories across these two distinct periods is essential for developing effective screening protocols. Adhering to a “wait-and-see” strategy until classical symptoms fully manifest at age 3 may overlook the optimal window for preventive intervention (10). This review aims to synthesize recent research progress on behavioral markers in infancy, explicitly differentiating between the subtle prodromal signs of the first year and the consolidating core symptoms of the second year. Furthermore, we explore the heterogeneity of these developmental trajectories and the potential of integrating behavioral observation with emerging screening technologies to optimize early identification strategies.

## 2 Core behavioral markers in infancy

Unlike the static diagnostic criteria applied to older children, behavioral markers in infancy manifest as deviations in developmental trajectories. These markers typically emerge in a specific temporal pattern, with subtle social-communication deficits often preceding the onset of RRBs.

### 2.1 Social communication deficits

Social communication impairments are the hallmark of ASD. Longitudinal studies utilizing home videos and prospective designs have mapped the trajectory of these deficits from the prodromal phase into the second year of life (11).

#### 2.1.1 Abnormalities in gaze and joint attention (JA)

Typically, JA evolves from responding (RJA, 6–9 months) to initiating (IJA, 9–12 months) (12, 13)*.* In ASD, this trajectory is often disrupted. Jones and Klin (14) utilized eye-tracking technology to reveal a critical “regressive” phenomenon: infants later diagnosed with ASD exhibited normal eye contact at 2 months but demonstrated a steady decline in fixation on the eyes from 2 to 6 months, accompanied by increased fixation on the mouth or body. By 12–24 months, deficits in both RJA and IJA become robust predictors of ASD. Unlike typically developing peers who coordinate gaze with gestures to share enjoyment, infants with ASD often fail to engage in these triadic interactions (child-object-caregiver).

#### 2.1.2 Diminished response to name

Responding to one's name is a fundamental social milestone usually achieved by 5 months (15). A diminished or absent response to name is one of the most consistent “red flags” in the first year. Miller et al. (16) conducted a prospective longitudinal study involving 156 infants (comprising ASD, high-risk, and low-risk groups) tested at 6, 9, 12, 15, 18, and 24 months. Their analysis confirmed that infants later diagnosed with ASD were significantly more likely to fail name-response tasks starting at 9 months, a deficit that persisted through 24 months. Crucially, this failure is not due to auditory deficits but reflects a lack of social prioritization. Persistent failure to respond to name at 12 months is highly specific for ASD and predicts poorer receptive language outcomes at age 3.

#### 2.1.3 Deficits in imitation and social smiling

Imitation acts as a primary engine for social learning. While neurotypical infants imitate facial expressions and simple sounds as early as 2–3 months, and accurate instrumental actions by 9–12 months (17), infants with ASD show marked delays (18). Poon et al. (19) analyzed coded home videos of 29 children later diagnosed with ASD. Their quantitative results indicated that by 9–12 months, the imitation and play skills of ASD children were comparable only to those of typically developing infants aged 3–9 months. Furthermore, a reduced frequency of “social smiles” (smiling directed at people rather than objects) during the first year serves as a subtle but significant indicator of reduced social reward processing (20).

### 2.2 Pre-linguistic communication delays

Before the onset of spoken language, communication deficits in ASD are already evident in the absence or atypicality of compensatory non-verbal strategies. Gestural communication acts as a critical bridge in this developmental phase. Liu et al. (21) conducted a longitudinal semi-structured assessment on 47 high-likelihood (EL) and 27 low-likelihood (LL) infants aged 9–19 months to code gesture frequency, communicative function, and integration. Their findings revealed that the development of JA gestures in the EL group significantly lagged behind the LL group, with developmental trajectories beginning to diverge at 14–18 months. Specifically, infants meeting the diagnostic threshold for ASD showed a reduction in social interaction gestures at 12–13 months, fewer gestures combined with communication skills (Gesture-Manner integration) at 15–16 months, and fewer gestures combined with eye contact (Gesture-Eye integration) at 18–19 months. Importantly, overall gesture frequency and Gesture-Manner integration were significantly correlated with scores on the Autism Diagnostic Observation Schedule (ADOS).

A further key differentiator in the second year (12–24 months) is the specific deficit in proto-declarative gestures (e.g., pointing to share interest), while proto-imperative gestures (e.g., reaching to request needs) remain relatively preserved (22, 23).

### 2.3 Vocalizations

The speech communication impairment in ASD represents a systemic disintegration of social communication function rather than a simple lag in language skills, manifesting as multidimensional abnormalities in language development, form, function, and social application (18). During the prodromal phase (6–12 months), infants with ASD may produce fewer canonical babbles (consonant-vowel combinations) and exhibit atypical vocalizations such as high-pitched squeals or growls (24). Clinically, this presents as a significant reduction in social vocalizations between 6 and 12 months. By 16 months, there is often a lack of stable, meaningful words; by 18–24 months, vocabulary size is typically less than 10 words with no spontaneous two-word combinations. Speech, when present, is frequently characterized by immediate echolalia rather than communicative intent (25).

Regarding the neural mechanisms underlying these deficits, Romeo et al. (26) demonstrated through a prospective longitudinal study that 18-month-old high-risk children—particularly those later diagnosed with ASD—may be cognitively and neurally more sensitive to their language environment. Their findings suggest that the neural oscillatory mechanisms mediating input-language associations differ significantly between children who develop ASD and those who do not (27).

### 2.4 RRBs

RRBs, defined as non-functional, highly repetitive, and difficult-to-interrupt motor, vocal, or sensory behaviors, are a core diagnostic criterion for ASD (28). While social deficits may be detectable in the first year, classic RRBs often manifest slightly later or evolve from lower-order motor abnormalities (29).

In the 12–24 month window, “lower-order” motor stereotypies become prominent, including hand flapping, body rocking, and posturing (30). Before the emergence of complex rituals, infants may exhibit prolonged visual fixation on geometric patterns, spinning objects, or lights. This phenomenon of “sticky attention” (difficulty disengaging visual attention) appearing before 12 months is strongly associated with the severity of later ASD symptoms (14). Unlike the “insistence on sameness” seen in older children, toddler-age RRBs are predominantly sensorimotor in nature.

## 3 Early regulatory and sensory features

While social communication deficits define the diagnosis of ASD, non-social features are often the earliest “red flags” perceived by caregivers. These domain-general impairments in attention, sensory processing, and physiological regulation may represent underlying neural endophenotypes that precede the emergence of core social symptoms.

### 3.1 Motor delays and abnormalities

Recent evidence-based research suggests that motor delays and functional abnormalities are among the earliest developmental deviations in ASD, often preceding social communication deficits in some high-risk infants. Longitudinal cohort studies have shown that high-risk infants may exhibit head lag on pull-to-sit tasks, delays in gross motor milestones (e.g., sitting, crawling, walking), and fine motor differences as early as 6 months, a time when social communication skills are not yet significantly affected (31). A recent prospective study on Chinese high-risk infants further confirmed that gross motor scores in the ASD group were significantly lower than those of other groups at 6–9 months.

Notably, motor development is not merely a linear lag. Through Latent Class Trajectory Modeling (LCTM), researchers discovered that approximately 11.6% of high-risk infants manifest a unique “fluctuating trajectory.” This pattern is characterized by initial improvement in motor skills followed by significant regression between 18 and 24 months (32). This regression in gross motor development is highly correlated with a final ASD diagnosis, whereas traditional single-point assessments often overlook these dynamic pathological changes.

Moreover, subtle motor asymmetries and deficits in postural control may limit an infant's ability to explore the environment and use gestures, thereby indirectly impeding the development of social communication (33). Further analysis reveals that early motor development indicators are significantly correlated with language and social skills at 24–36 months (34), suggesting that motor abnormalities serve not only as early warning signals but may also participate in the shaping of social communication pathways. Therefore, longitudinal motor monitoring of high-risk infants starting from 6 to 9 months holds significant clinical value. This approach not only aids in early intervention and risk assessment but also facilitates the identification of ASD subtypes with specific developmental trajectories.

### 3.2 Sensory processing abnormalities

Sensory features refer to an individual's ability to perceive, regulate, and respond to environmental sensory stimuli (e.g., sound, light, touch). Clinically, sensory abnormality patterns in ASD are primarily categorized into hypo-reactivity, hyper-reactivity, sensory seeking, and enhanced perception (35). Given their prevalence and specificity, the DSM-5 has formally included “hyper- or hyporeactivity to sensory input” as a core diagnostic feature of ASD (11)*.*

In recent years, the research focus has shifted from mere phenomenological description to mechanistic exploration. Hadad et al. (36), through a systematic review, posited that atypical sensory perception is not just a phenotypic marker of ASD but a fundamental characteristic of the autistic psyche (37). Their research model reveals that individuals with ASD show reduced “empirical learning” for both social stimuli (e.g., faces, voices) and non-social stimuli (e.g., orientation), suggesting widespread alterations in underlying perceptual mechanisms (38). Crucially, these signs of perceptual abnormality appear as early as 9–10 months, often preceding overt social symptoms. Based on this, researchers advocate for combining psychophysical measurements with computational models as a primary step to decode the perceptual characteristics of ASD. By revealing potential neural computational processes and their developmental pathways, there is hope for early intervention strategies that correct deviant perceptual-behavioral trajectories, realizing a shift from “symptom management” to “mechanistic intervention.”

### 3.3 Attentional disengagement (“sticky attention”)

Attentional disengagement—the ability to shift focus from one stimulus to another—is crucial for navigating a dynamic social environment. A phenomenon known as “sticky attention” is frequently observed in infants at high risk for ASD. Research indicates that 12-month-old infants who are later diagnosed with ASD exhibit significantly prolonged latencies in disengaging visual attention from central stimuli (39, 40). This is not merely an attentional quirk but a mechanistic barrier to social learning: if an infant is “stuck” on a geometric object or a light source, they miss the fleeting social cues (e.g., a mother's gaze or smile) occurring in the periphery. This early “attentional tunnel vision” may initiate a cascade of deprivation in social input, thereby exacerbating deficits in cortical specialization (41).

### 3.4 Sleep-wake rhythm disturbances

Sleep disturbances constitute one of the earliest physiological indicators of CNS anomalies in ASD, affecting 50%–80% of cases—nearly double the rate in typically developing peers (42). Beyond prevalence, the longitudinal trajectory of these disturbances offers superior predictive value over static observations. A recent large-scale analysis of the JECS cohort (*n* = 63,418) by Kikuchi et al. (43) identified symptom persistence during the first year as the critical risk factor; unlike transient infant sleep issues, early-onset and persistent difficulties significantly elevated the risk of an ASD diagnosis at age 3.

Evidence increasingly suggests a bidirectional exacerbation mechanism between sleep and core symptoms. Longitudinal neuroimaging indicates that sleep onset difficulties in high-risk infants diverge from typical trajectories as early as 6–12 months. MacDuffie et al. (44) demonstrated that these early disruptions predict altered hippocampal development and subsequent symptom severity. Bridging biology and behavior, Begum-Ali et al. (45) recently delineated a specific pathway: poor night sleep quality in infancy (particularly by 14 months) predicts disrupted “social attention” trajectories, which in turn mediate later ASD traits. Mechanistically, sleep deprivation may aggravate neurodevelopmental outcomes by disrupting synaptic homeostasis and the consolidation of social visual experiences during critical periods (46).

### 3.5 Emotion dysregulation

Emotion Dysregulation (ED) often becomes part of the ASD phenotype by 12–24 months (47), with early manifestations including intense emotional reactions, irritability, difficulty being soothed, and overreactivity to environmental changes. As a severe manifestation of ED, Self-Injurious Behavior (SIB) occurs at a significantly higher rate in children with ASD compared to the general population. Unlike in adolescence, SIB in infancy primarily manifests as head banging, self-hitting, or skin biting (48).

Dimian et al. (49) conducted a prospective longitudinal assessment of 235 infants at high risk for ASD. Their logistic regression analysis demonstrated that the presence of SIB at 12 months (e.g., head banging), combined with low developmental levels, served as a robust predictor of both an ASD diagnosis and deficits in adaptive functioning at 24 months. These findings underscore the importance of incorporating emotion regulation and SIB into early screening dimensions, alerting clinicians to these early warning signs so that behavioral interventions can be initiated before symptoms crystallize.

## 4 Developmental trajectories and heterogeneity

A major challenge in early identification lies in the significant heterogeneity of symptom onset and progression. Current evidence posits that ASD is not a static condition that simply “emerges,” but rather a dynamic process of deviation from normative developmental pathways. Understanding these distinct trajectories is crucial for minimizing false negatives in early screening (50).

### 4.1 Distinct onset patterns: early onset vs. regressive

**Figure 1:**
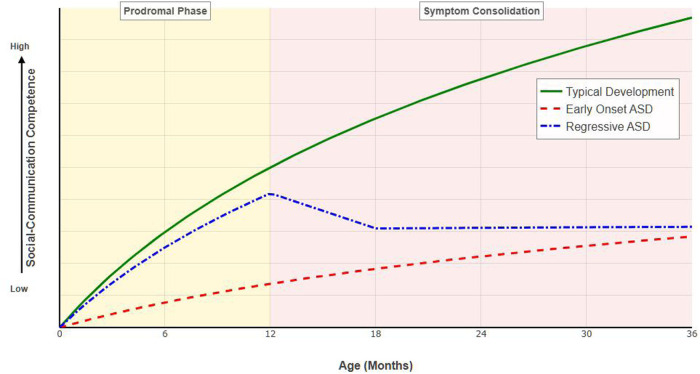
This schematic illustrates distinct developmental patterns of social-communication competence over the first 36 months of life. Typical development (green solid line): represents the normative acquisition of social milestones, reaching a standardized competence level by 36 months. Early-onset ASD (red dashed line): characterized by profound delays evident from early infancy, reflecting a “failure to acquire” skills pattern. Regressive ASD (blue dash-dot line): depicts a trajectory where infants may gain early skills—often with subtle, sub-threshold delays (lower slope than typical)—during the prodromal phase (0–12 months, yellow shading), followed by a gradual loss or plateauing of skills during the symptom consolidation phase (12–36 months, red shading). Adapted from Landa et al. (51, 52).

Retrospective and prospective studies delineate at least two distinct behavioral phenotypes regarding symptom onset (Figure 1). The first is the early-onset pattern, observed in approximately one-third of infants, who exhibit social and sensorimotor deviations—such as a persistent lack of visual fixation or motor delays—very early in life (0–6 months). These infants appear to demonstrate a “failure to acquire” skills from the outset. The second is the regressive pattern, affecting a substantial proportion of children (estimated between 20% and 40%). These infants may achieve typical social milestones, such as babbling and eye contact, during the first year but subsequently experience a developmental regression or plateauing between 15 and 24 months (53). Crucially, recent evidence suggests that this regression is often gradual rather than sudden. The perceived “loss” of skills is frequently preceded by subtle, sub-threshold delays in the first year. Consequently, a child who passes screening at 12 months but fails at 24 months likely represents a specific developmental trajectory rather than a failure of the screening instrument.

### 4.2 The “prodromal” phase and developmental cascades

**Figure 2:**
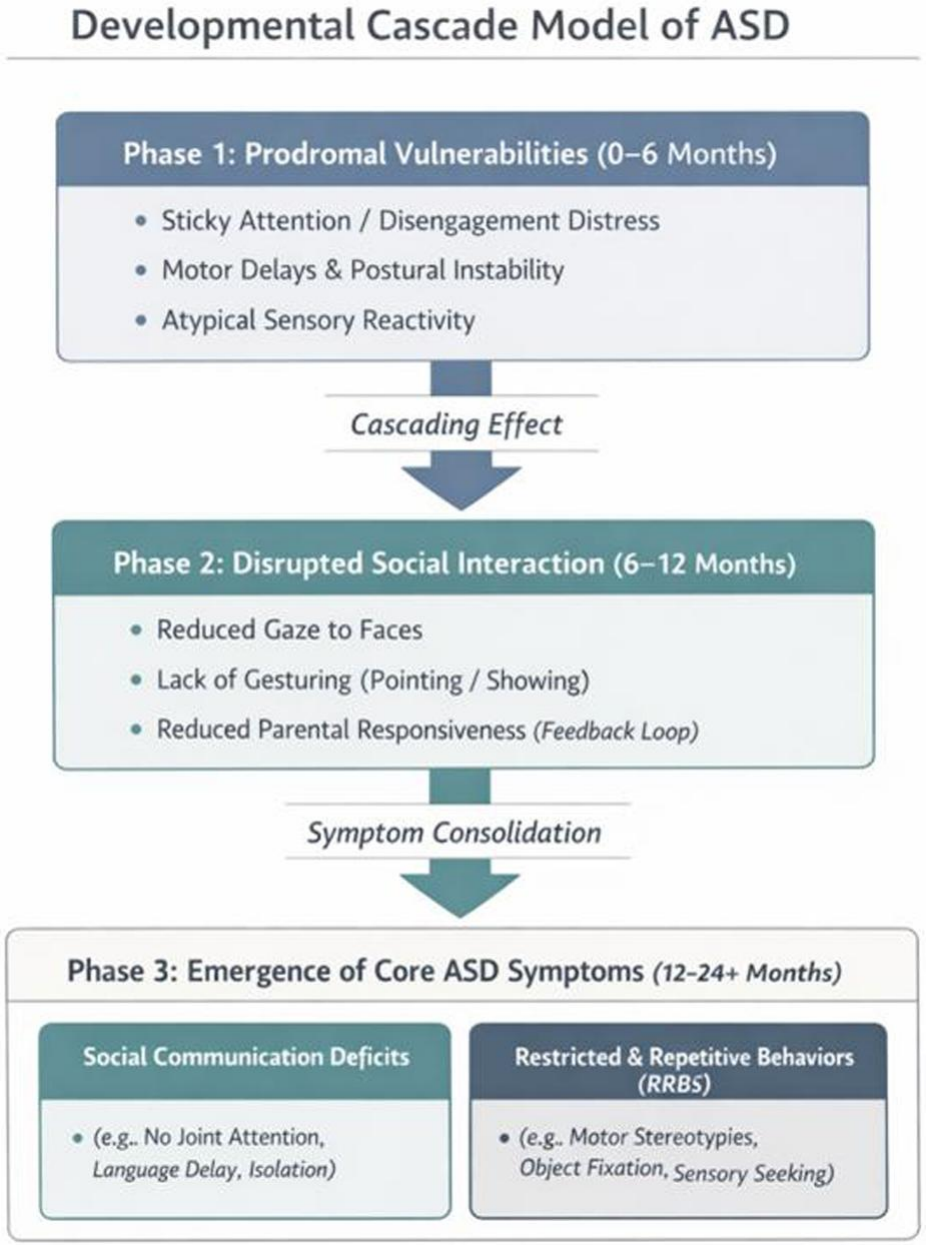
The developmental cascade model of ASD. This conceptual framework illustrates how early, domain-general vulnerabilities in the first 6 months of life (Phase 1, e.g., sticky attention, motor delays) trigger a downstream chain reaction. These primary deficits hinder the infant's ability to engage with the environment, leading to disrupted social interactions and attenuated parental responsiveness during the second half of the first year (Phase 2). Through this dynamic transaction between the infant and the environment, these initial deviations gradually consolidate into the core diagnostic behavioral phenotype of ASD—comprising social communication deficits and restricted, repetitive behaviors (RRBs)—by the second year of life (Phase 3). Adapted from Bradshaw et al. (33).

The period from 0 to 12 months is best conceptualized as a prodromal phase, characterized by non-specific vulnerabilities rather than core diagnostic features. The theory of “Developmental Cascades” suggests that early, low-level deficits trigger a chain reaction affecting higher-level functions (33) (Figure 2).

For instance, primary deficits in motor control (e.g., postural instability) may limit an infant's ability to free their hands for gesturing (34). This lack of gesturing reduces parental responsiveness, which in turn diminishes language input, ultimately culminating in social communication deficits. This model underscores that “autism symptoms” are the downstream consequences of altered infant-environment interactions over time.

### 4.3 Impact of environmental factors

While ASD has a strong genetic basis, the trajectory of symptom severity is modulated by environmental factors. High-risk infants may be biologically less equipped to actively seek social input; if the environment fails to provide compensatory stimulation (e.g., reduced dyadic interaction), symptoms may be exacerbated. Furthermore, emerging evidence suggests a correlation between excessive early screen time and ASD-like symptoms in susceptible infants. While screen exposure does not “cause” ASD, it may displace critical opportunities for social learning, potentially amplifying social withdrawal in children with pre-existing neurodevelopmental vulnerabilities (54).

## 5 Advances in early screening tools and future directions

Despite the identification of numerous prodromal markers, translating these findings into clinical practice remains a challenge. Current screening paradigms are often “one-size-fits-all,” failing to capture the heterogeneity described above. The Modified Checklist for Autism in Toddlers, Revised (M-CHAT-R/F) remains the gold standard for population-level screening; however, its sensitivity drops significantly when applied to children under 16–18 months (55). These tools rely heavily on parental reporting, which is subject to recall bias, and as static instruments, they often miss the subtle qualitative impairments characteristic of the prodromal phase (e.g., the *frequency* vs. *quality* of gaze).

To overcome the subjectivity of questionnaires, the field is moving toward Digital Phenotyping—the quantification of human behavior using digital devices. Automated eye-tracking technology can detect reduced fixation on social scenes as early as 6 months. Recent advancements have made these tools portable (e.g., tablet-based), allowing for scalable screening in primary care settings (56). Furthermore, Artificial Intelligence (AI) algorithms, including Machine Learning (ML) and Computer Vision, are being trained to analyze home videos. These systems can automatically code motor patterns (e.g., head lag), vocalizations, and facial expressions with a precision that exceeds human observation, offering a potential non-invasive biomarker for early risk assessment (57).

## 6 Conclusion

The clinical imperative for ASD is shifting from “diagnosing a disorder” at age 3 to “detecting developmental risk” in infancy. Rather than a sudden onset, ASD emerges through a developmental cascade rooted in the first year of life. Prodromal vulnerabilities—including motor delays, “sticky” attention, and sensory abnormalities—often predate overt social deficits. Recognizing the heterogeneity of these trajectories is paramount, particularly for “regressive” patterns where early milestones are transiently met.

Moving forward, the field must transcend static checklists in favor of longitudinal surveillance integrating behavioral observation with digital biomarkers. While universal screening remains essential for the general population, the intensive monitoring of these subtle prodromal markers is particularly critical for high-likelihood groups to facilitate symptom-based intervention. By identifying and intercepting deviant trajectories before core symptoms crystallize, we can leverage early neuroplasticity to fundamentally alter the long-term prognosis for affected children and their families.
